# ddRAD Sequencing-Based Identification of Genomic Boundaries and Permeability in *Quercus ilex* and *Q. suber* Hybrids

**DOI:** 10.3389/fpls.2020.564414

**Published:** 2020-09-04

**Authors:** Unai López de Heredia, Fernando Mora-Márquez, Pablo G. Goicoechea, Laura Guillardín-Calvo, Marco C. Simeone, Álvaro Soto

**Affiliations:** ^1^ G.I. Genética, Fisiología e Historia Forestal, Dpto. Sistemas y Recursos Naturales, ETSI Montes, Forestal y del Medio Natural, Universidad Politécnica de Madrid, Madrid, Spain; ^2^ Department of Forestry, NEIKER-BRA, Vitoria-Gasteiz, Spain; ^3^ Dipartimento di Scienze Agrarie e Forestali (DAFNE), Università degli Studi della Tuscia, Viterbo, Italy

**Keywords:** ddRADseq, *Quercus*, hybridization, introgression, genomic boundaries, SNPs

## Abstract

Hybridization and its relevance is a hot topic in ecology and evolutionary biology. Interspecific gene flow may play a key role in species adaptation to environmental change, as well as in the survival of endangered populations. Despite the fact that hybridization is quite common in plants, many hybridizing species, such as *Quercus* spp., maintain their integrity, while precise determination of genomic boundaries between species remains elusive. Novel high throughput sequencing techniques have opened up new perspectives in the comparative analysis of genomes and in the study of historical and current interspecific gene flow. In this work, we applied ddRADseq technique and developed an *ad hoc* bioinformatics pipeline for the study of ongoing hybridization between two relevant Mediterranean oaks, *Q. ilex* and *Q. suber*. We adopted a local scale approach, analyzing adult hybrids (*sensu lato*) identified in a mixed stand and their open-pollinated progenies. We have identified up to 9,251 markers across the genome and have estimated individual introgression levels in adults and seedlings. Estimated contribution of *Q. suber* to the genome is higher, on average, in hybrid progenies than in hybrid adults, suggesting preferential backcrossing with this parental species, maybe followed by selection during juvenile stages against individuals with higher *Q. suber* genomic contribution. Most discriminating markers seem to be scattered throughout the genome, suggesting that a large number of small genomic regions underlie boundaries between these species. In adult hybrids 273 markers (3%) showed allelic frequencies very similar to one of the parental species, and very different from the other; these loci could be relevant for understanding the hybridization process and the occurrence of adaptive introgression. Candidate marker databases developed in this study constitute a valuable resource to design large scale re-sequencing experiments in Mediterranean sclerophyllous oak species and could provide insight into species boundaries and adaptive introgression between *Q. suber* and *Q. ilex*.

## Introduction

The relevance of hybridization as an adaptive and evolutionary driving force is a controversial topic. Even the different concepts of species are tightly linked to the relevance authors concede to gene exchange among species (reviewed in Harrison and Larson, 2014). The Biological Species Concept (Mayr, 1982) relies mainly on reproductive isolation among species, while other perspectives admit the “porous” nature of genomes and stress ecological differentiation instead (f. i., Mallet, 2007). Actually, hybridization and introgression are common in plants, where interspecific gene flow is supposed to be an ongoing process in approximately 25% of species (Mallet et al., 2016). Hybridization is especially frequent in certain taxa, as is the case of *Quercus* species, which have been proposed as models for the Ecological Species Concept (Burger, 1975; van Valen, 1976), and are commonly regarded as “syngameons” (Cannon and Petit, 2020; Kremer and Hipp, 2020). Temperate oaks from Europe and North America have become references for hybridization studies and they have shown that hybridization might be crucial to understand their successful evolutionary trajectories (f.i., Petit et al., 2003; Lepais et al., 2009; Eaton et al., 2015; Goicoechea et al., 2015; Ortego et al., 2018; Crowl et al., 2019).

Hybridization can allow one species to incorporate genes or alleles from another one. Some of these transferred genes can confer a selective advantage to the first species, improving its performance or increasing its ecological range (Sexton et al., 2009). This process is known as adaptive introgression, recently reviewed for plants by Suárez-González et al. (2018). Human-mediated hybridization has been largely used in agriculture, and analysis of spontaneous introgression to search for useful adaptations for crop breeding has been recently claimed, for instance, by Burgarella et al. (2019). Additionally, hybridization has probably played a key role in plant species demography. Threats to small populations can be intensified by lower individual fitness (Allee effect), including reproductive success, maybe related to inbreeding depression. Recurrent hybridization can increase effective population size, overrunning this effect. Moreover, hybridization can also act as a colonization mechanism over other species range (Potts and Reid, 1988; Petit et al., 2003).

Notwithstanding this ample capacity of interspecific gene flow, *Quercus* species still maintain their integrity (f.i., Valbuena-Carabaña et al., 2005; Moran et al., 2012). Cannon and Scher (2017) presented a well-constructed hypothesis accounting for this apparent paradox. The authors focused on the behavior of male hybrid gametes in the diploid stigma of a pure individual of one of the parental species. In such a situation, it is argued that hybrid gametes most similar to those of that parental species have advantage over the other hybrid gametes. Thus, resulting backcrosses would be quite similar to parental species, bearing, at maximum, a low proportion of introgressed alleles. This would be the reason why species boundaries do not get blurred so often even when there is hybridization. However, such hypothesis does not consider the opposite situation, with hybrids acting as mother trees. In this case, in order to parallel Cannon and Scher hypothesis, it would not be male gametes (assumed to come from parental species), but the female gametes and/or the almost pure zygotes which should show better fitness on a hybrid ovary.

*Quercus ilex* (holm oak) and *Q. suber* (cork oak) constitute a very suitable experimental system for hybridization analysis. These two Mediterranean species are easily distinguishable due to their phenotype, mainly according to the characteristic corky bark of *Q. suber.* Hybridization between both species is well known since ancient times, but it is considered to be rather infrequent. Therefore, their introgression should be more easily traceable than, for instance, the more frequent hybridization and introgression between white oaks, which often hampers correct identification of individuals (Muir et al., 2000; Valbuena-Carabaña et al., 2005). Hybridization is supposed to have played a key role in the survival of *Q. suber* in Southern France and Eastern Spain, in a general landscape of calcareous soils, not suitable for *Q. suber* (Lumaret et al., 2002; Jiménez et al., 2004; López de Heredia et al., 2007). In 2009, current hybridization was estimated in less than 2%, in a study using eight nSSR and including natural pure and mixed stands, as well as provenance trials covering the whole distribution range of *Q. suber* (Burgarella et al., 2009). Nevertheless, recent studies have shown that classification/identification power of the set of nSSR used is too low, and thus, hybridization and introgression could have been underestimated (López de Heredia et al., 2018a; Soto et al., 2018). Therefore, more powerful marker sets are needed in order to obtain accurate estimations.

In recent years, rapid advancement and affordability of next generation sequencing (NGS) methodologies have largely increased the scope and accuracy of genome-wide analysis and its application in phylogenetics, adaptation, hybridization, and breeding studies. For oak species, significant efforts have been made in the white oak complex, producing valuable genomic resources: ESTs, QTLs, SNP panels, or linkage maps, among others (Saintagne et al., 2004; Ueno et al., 2010; Lepoittevin et al., 2015; Lesur et al., 2015; Bodénès et al., 2016; Lesur et al., 2018; Lang et al., 2020). The ongoing progress in NGS technologies has resulted in a first draft genome assembly of *Q. suber* (Ramos et al., 2018), and in a more complete assembly for *Q. robur* (Plomion et al., 2018). The latter is the first oak genome assembly that contains information at the chromosome level, thus easing comparative genomic analysis in less studied oak species (see for instance Goicoechea et al., 2015).

The unavailability of reference genome assemblies constrains the scope of genomic studies in non-model species, as is the case for the vast majority of woody plant species (Falk et al., 2018). In this context, the use of reduced representation genotyping methodologies, such as double-digested RAD sequencing (ddRADseq; Peterson et al., 2012), largely facilitates genomic analysis (Hirsch et al., 2014), and has been already used in the phylogenetic analysis (Hipp et al., 2020) and genetic mapping of oak species (Konar et al., 2017). The use of these methodologies, together with the aforementioned NGS-based genomic resources and specifically designed bioinformatic pipelines, opens up new perspectives in oak species genomics.

Here we report the application of ddRADseq to the study of introgression and ongoing hybridization between *Q. ilex* and *Q. suber*, using adult hybrids identified in a natural population and their progenies, since natural hybrids can be quite useful for mapping differences and reproductive barriers in non-model organisms (Baack and Rieseberg, 2007). The specific aims of the study were: (1) to identify candidate markers for the study of ongoing hybridization and adaptive introgression and (2) to provide insight into the reproductive behavior of hybrids as mother trees.

## Materials and Methods

### Plant Material and DNA Extraction

The sampling area is located in Fregenal de la Sierra (Badajoz province, southwestern Spain). Twenty-two adult hybrid individuals were identified according to morphological characters, especially those regarding bark, described by Natividade (1936). Leaves from these hybrids, along with leaves from 98 *Q. suber* and 99 *Q. ilex* adult individuals were collected and stored at −80°C for further DNA extraction.

In addition, in November 2016, we collected acorns from nine of these hybrid trees, eight of them located in the same estate, in a mixed *Q. ilex-Q. suber* stand. A total of 1,270 acorns (492 from hybrids, 397 from four cork oaks, and 381 from four holm oaks, presumably unrelated) were collected directly from the canopies, so as to be sure of the identity of mother trees. Acorns were first sown on perlite and seedlings were transplanted to 3 l. containers, in peat:perlite (3:1), and grown in nursery conditions. Hybrid acorns showed lower germination rate, so that the final sampling size for DNA extraction and genotyping consisted of 302 hybrids (22 adults and 280 seedlings), 467 cork oaks (98 adults and 369 seedlings), and 439 holm oaks (99 adults and 340 seedlings) (**Table 1**).

**Table 1:** Number of seedlings in open-pollinated families, identified by the name of each mother tree.

	No. of seedlings		No. of seedlings
*Quercus ilex*		*Hybrids*	
E28	82	FS08	12
E31	84	FS14	19
E41	81	FS16	47
E96	93	FS17	26
*Quercus suber*		FS18	16
A05	71	FS19	57
A07	94	FS20	30
A09	101	FS21	5
A10	103	FS22	68

Leaf discs (6 mm diameter) from adults and seedlings (N = 1,208 samples) were used for DNA extraction on a Genespin™ platform, and for further library preparation and sequencing (LGC Genomics GmbH, Germany).

### Identification of Hybrids’ Plastid Lineages

The trnH-psbA intergenic spacer of chloroplast DNA was amplified and sequenced following Simeone et al. (2016) in the four *Q. suber* and the four *Q. ilex* from which acorns were sampled, the 22 adult hybrids and 18 of the hybrid progeny individuals. Four additional adult hybrid individuals from Central Spain (López de Heredia et al., 2018a) were also analyzed. Electropherograms were edited and eye-checked with Chromas 2.6.2 (http://technelysium.com.au). The obtained dataset was enriched with all GenBank accessions showing 100% identity in a nucleotide BLAST search on NCBI (https://www.ncbi.nlm.nih.gov/), and with the trnH-psbA haplotypes representing all the known evolutionary lineages of the West Eurasian *Quercus* sections *Ilex* and *Cerris* (Simeone et al., 2016; Vitelli et al., 2017; Simeone et al., 2018). A multiple alignment was built with MEGA7 (Kumar et al., 2016) and a haplotype list was computed with DnaSP 5.1 (Librado and Rozas, 2009). A median-joining (MJ) haplotype network of the entire dataset was inferred with Network 4.6.1.1 (http://www.fluxus-engineering.com/). The MJ algorithm was invoked with default parameters (equal weight of transversion/transition), treating gaps as 5th state.

### ddRADseq Library Construction and Sequencing

For ddRADseq analysis of all samples, a pilot study was conducted using the protocol implemented on RADdesigner (Guillardín-Calvo et al., 2019) to assess the optimal enzyme combination, type of library, and number of reads per sample for the aims of the study. Based on this pilot study, PstI/MspI were selected as restriction enzymes. The use of methylation-sensitive restriction enzyme combinations such as PstI/MspI increases the fraction of coding DNA among the sequenced fragments, which is of particular interest in complex plant genomes (Pootakham et al., 2015). ddRADseq libraries were constructed and sequenced by LGC Genomics GmbH (Germany), including a barcode to identify the fragments corresponding to each sample. Libraries were prepared in 14 96-well PCR plates, with 96 inline-barcoded adaptors, and 10 ul of each library were amplified using MyTaq (Bioline GmbH, Germany) and TrueSeq primers (Illumina, USA) in 20 ul PCR reactions. Five ul of each amplified library from the same PCR plate were pooled and cleaned up using Agencourt AMPure XP bead purifications (Beckman Coulter, USA) and MinElute columns (QIAGEN, Netherlands). Normalization was done using Evrogen Trimmer kit (Evrogen, Russia) and reamplified in 100 ul using MyTaq (Bioline GmbH, Germany). Two hundred to 400 bp fragments were selected on Blue Pippin and LMP-agarose gel, in order to produce fewer, yet sufficient, fragments at higher depth. Sequencing was performed on an Illumina NextSeq 500 V2 platform (Illumina Inc., USA) and several runs were performed in order to obtain ~1.5 M 150 bp single-ended reads per sample.

### Bioinformatic Analysis

**Figure 1:**
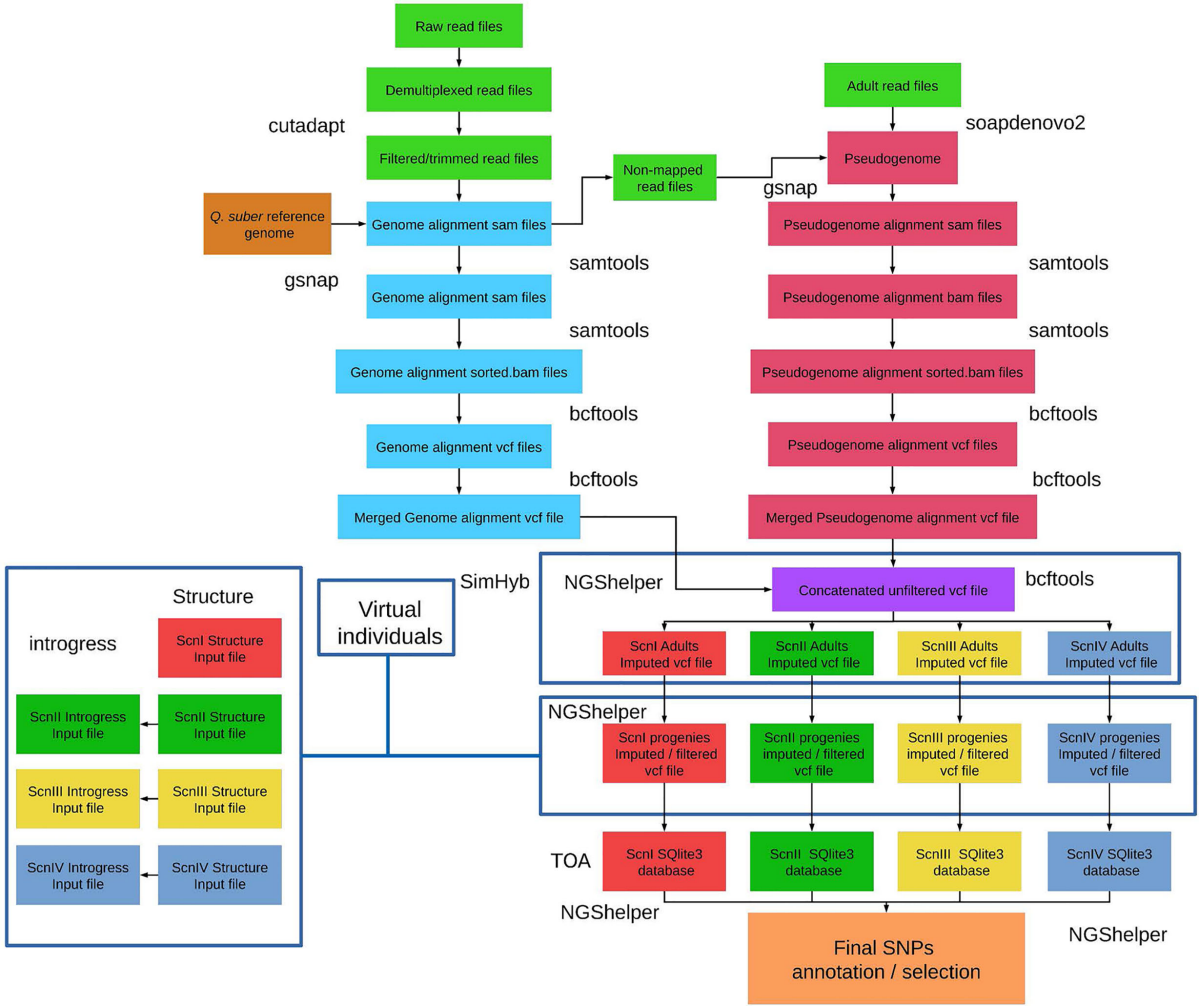
Bioinformatics strategy for ddRADseq data analysis. BASH/R scripts and instructions to install software and dependencies are available by downloading the ddRAD-CORK-HYB package at https://github.com/GGFHF/ddRAD-CORK-HYB.

All the scripts to perform the bioinformatic analysis detailed below (**Figure 1**) are stored in the ddRAD-CORK-HYB package (https://github.com/GGFHF/ddRAD-CORK-HYB). A README file provides instructions to install software and dependencies, and includes information about each script parameter and how to run scripts properly.

#### Read Pre-Processing and Filtering

Read preprocessing consisted in a demultiplexing step of all library groups using the Illumina BclFastQC 2.17.1.14 software (http://support.illumina.com/downloads/bcl2fastqconversion-software-v217.html). A maximum of two mismatches or Ns in the barcode read for demultiplexing between libraries was set when the barcode distances between all libraries on the lane allowed for it. Demultiplexing of library groups into samples was performed according to their inline barcodes. No mismatches or Ns were allowed.

Filtering was performed with cutadapt (Martin, 2011), and sequencing adapter remnants were clipped from all reads. Reads with final length <20 bp and reads with 5′ ends not matching the restriction enzyme site were discarded. Finally, reads were filtered by quality, removing reads containing Ns and trimming those reads at the 3′-end to get a minimum average Phred quality score of 20 over a ten-base window. Again, reads with a final length <20 bp were discarded. Quality of pre-processed read files was checked with FastQC (Andrews, 2010).

#### Read Alignment and Variant Calling

Gsnap (Wu et al., 2016) was used to align pre-processed and filtered reads to the genome assembly of *Q. suber* (Ramos et al., 2018). Reads that did not map to the *Q. suber* genome assembly were aligned back with Gsnap to a pseudogenome built with SoapDeNovo2 genomic assembler (Luo et al., 2012) using the reads from the adult individuals, and filtered by size to keep only contigs >100 bp.

Genome and pseudogenome alignments in *sam* format were converted into compressed *bam* files and sorted with samtools (Li et al., 2009). Variant calling was performed individually with samtools *mpileup* and bcftools (Li, 2011) *call* options. Variant calling files of each sample from genome and pseudogenome alignments were concatenated with bcftools to obtain individual *vcf* files. Finally, all samples were merged with bcftools *merge* to obtain a single concatenated-merged unfiltered variant calling file for all the samples (adults, progenies, and replicates).

#### Determination of Marker Positions in *Q. robur* Linkage Groups

Multi-fasta sequences of the flanking regions 200 bp to each side of the selected loci positions were extracted from the *Q. suber* reference genome assembly and from the pseudogenome using the *get-flanking-regions.py* utility from NGShelper (https://github.com/GGFHF/NGShelper). The resulting multifasta file was subjected to a nucleotide BLAST search against *Q. robur* v. PM1N genome assembly with the local version of BLAST+ (Camacho et al., 2009) using an e-value threshold of 1E-6, to obtain the genomic positions in the *Q. robur* genome of the identified markers.

Further, we built a linear function to correlate the genomic coordinates in bp of the SNPs used to build linkage groups in *Q. robur* (Bodénès et al., 2016) with the positions of those SNPs in 12 *Q. robur* linkage groups in cM. All this information was freely available at the oak genome sequencing web (http://www.oakgenome.fr) and at the *Quercus* portal (https://arachne.pierroton.inra.fr/cgi-bin/cmap/). Thus, we were able to identify the putative location of our marker loci in the linkage groups of *Q. robur*, and to visualize them with the R package LinkageMapView (Ouellette et al., 2017).

#### Marker Databases

SQlite3 databases for each imputation scenario (see below) were constructed with NGShelper including information parsed from *vcf* files (type of marker, genomic coordinates, etc.) and functional annotation of selected markers. Functional annotations of the markers identified in the genome were collected from *Q. suber* genome *gff* files and descriptions of gene IDs was obtained from NCBI ‘All_Plants.gene.info’ file. The markers corresponding to the pseudogenome were annotated with Toa (Mora-Márquez et al., 2020), through nucleotide BLAST searches against NCBI NT databases.

#### Two-Step Imputation/Filtering Procedure: Scenarios

Missing data must be considered for *vcf* files processing steps. There is no standard missing data cutoff for excluding loci, and filtering criteria need to be established depending on the aims of the study (Huang and Knowles, 2016). Excessive data filtration can have unforeseen consequences, such as the truncation of loci with higher mutation rates or missing relevant loci. Besides, filtering protocols must ensure sufficient depth coverage of the remaining loci (Eaton et al., 2017). A common practice is to test for different scenarios encompassing more restrictive and more relaxed filtering criteria.

The procedures to perform filtering and imputation to account for missing data and/or null alleles are implemented in NGShelper. Input data consist of the single concatenated-merged unfiltered variant calling file generated in the previous step. The basic procedure included two steps. Firstly, the proportion of missing data in adult individuals of each parental species was considered. For loci with a low proportion of missing data in one of the parental species (md < 0.05) but with a high proportion in the other (md > 0.90), a null allele was imputed to missing data of the adult individuals of the latter species. Successive filtrations of imputed loci were performed, resulting in different imputation scenarios (see *Results* section). In a second step, genotypes of the seedlings were corrected according to their mother tree imputation state.

#### Estimation of Introgression Levels

Estimation of the introgression level of adult individuals and progenies was performed using the Bayesian approach implemented in Structure v. 2.3.4 (based on Pritchard et al., 2000), as well as by means of the hybrid index provided by Introgress (Gompert and Buerkle, 2010). Firstly, we checked the performance and accuracy of both classification methods using virtual individuals with known introgression levels. These individuals were simulated with SimHyb (Soto et al., 2018; https://github.com/GGFHF/SimHyb), based on the allele frequencies of the adult *Q. ilex* and *Q. suber* populations. Later on, we estimated contribution of parental species to the genome of each individual in two scenarios of prevalence of hybrids: 1 and 10%. In both cases, additional virtual *Q. ilex* and *Q. suber* individuals were included as reference. Number of real and virtual individuals used, according to their introgression level, are provided in Supplementary Information (**Supplementary Table 1**). In all the cases, we allowed Structure to estimate admixture proportions in the unknown samples. For all jobs, we used 10,000 burn-in steps followed by 100,000 repetitions of the MCMC chains, and the number of populations was set to K = 2, following the methodology described by Evanno et al. (2005).

## Results

### Adult Hybrid Identification and Germination Rates

Twenty-two adult individuals, identified as *Q. ilex–Q. suber* hybrids according to their phenotypic characters, were localized in the field, in different estates in the south of Badajoz (SW Spain). Acorns were collected from the canopy of these hybrid trees, as well as from unrelated *Q. ilex* and *Q. suber* adults (four of each species). While parent species showed high germination rates (89.24% for *Q. ilex* and 92.95% for *Q. suber*), only 56.92% of hybrid acorns germinated successfully.

**Figure 2:**
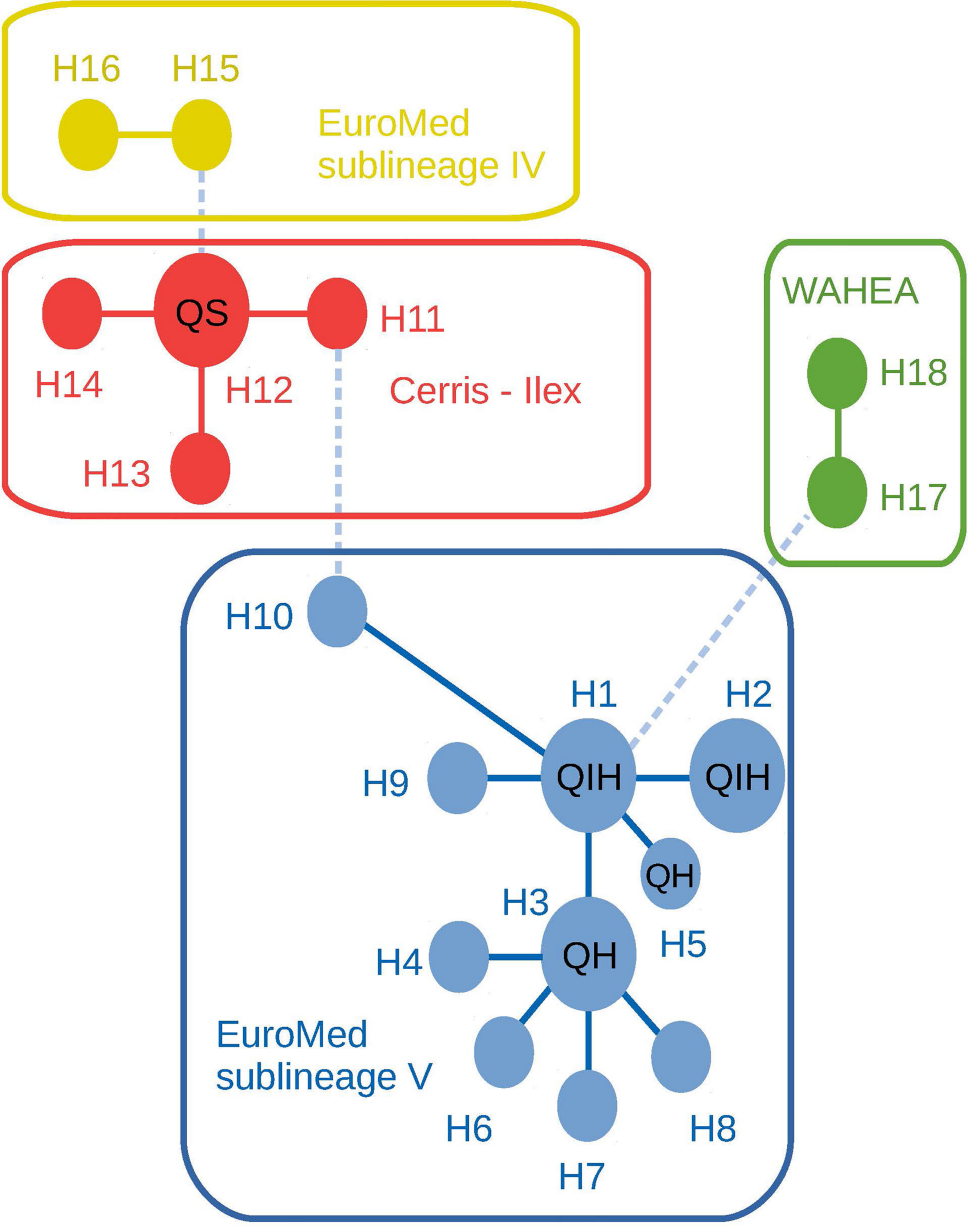
Median-joining haplotype network of trnH-psbA marker combining the investigated samples and published haplotypes of each major *Ilex* and *Cerris* lineages. Lineage and sub-lineage identity according to Vitelli et al., 2017 and Simeone et al., 2018. WAHEA: haplotypes from the West Asian, Himalayan, and East Asian lineages. Line types correspond to the number of mutations separating each haplotype: solid lines = 1–3 mutations; dashed lines 6–10 mutations. Coding within haplotype circles indicate the presence of samples of the present work: QS, Adult *Q. suber* individuals; QIH, Adult *Q. ilex* and hybrid individuals; QH, only hybrid individuals.

Chloroplast DNA analysis of these trees revealed that the four cork oak adult individuals gathered in a single haplotype (H12), corresponding to a widespread, likely ancestral haplotype, shared by multiple *Cerris* section species extending from Anatolia to the central Mediterranean. The four holm oak adult samples grouped with all hybrid adults and progenies in the ‘Euro-Med’ haplotype group (sub-lineage V) (Simeone et al., 2018), i.e. an exclusive lineage of the central and western Mediterranean members of section *Ilex* (*Q. ilex* and *Q. coccifera*) (**Figure 2** and **Supplementary List 1**). This result confirms the directionality in *Q. ilex–Q. suber* hybridization, in which holm oak acts as mother tree, and therefore hybrids carry holm oak chloroplasts. The trnH-psbA haplotype sequences generated in this study are available on GenBank under accession numbers LR797857-LR797862.

### Read Alignment, Variant Filtering, and Imputation

**Figure 3:**
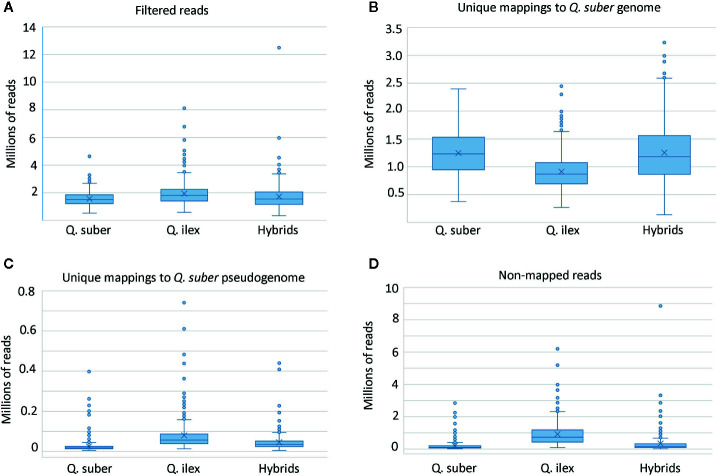
Box and whisker plots of mapping sequences. **(A)** Total number of reads employed in the ddRADseq pipeline after adapter removal and quality filtering. **(B)** Number of reads uniquely mapped to *Q. suber* genome assembly (Ramos et al., 2018). **(C)** Number of reads uniquely mapped to the pseudogenome generated with SoapDeNovo2. **(D)** Final number of non-mapped reads.

A mean number of 1.74 million single-ended reads per sample was obtained after removal of adapters and read filtering by quality. Sequences are available at NCBI SRA database (BioProject: PRJNA628590). From these, 64.9% of all filtered reads mapped uniquely to the *Q. suber* genome assembly (**Figure 3**). However, we found noticeable differences in the total number of aligned reads among progenies and adult individuals of each species. As expected, *Q. suber* libraries aligned better than *Q. ilex*, while the hybrids showed intermediate behavior. Reads showing ambiguous mapping were discarded, and reads that did not map to the *Q. suber* genome assembly were aligned to the pseudogenome, improving total alignment percentages to 67.8% of all filtered reads. The best alignments to the pseudogenome were obtained for those *Q. ilex* libraries with the poorest mapping to the *Q. suber* genome.

After individual variant calling, the number of variants ranged between 14,593 and 524,458 for the genome and between 212 and 66,680 for the pseudogenome alignments. The final concatenated-merged variant calling file had 16,234,798 variants, of which 97.4 % were SNPs and <5% multi-allelic sites. This massive *vcf* file was subjected to filtering and imputation according to four scenarios.

As a basic filtering, we removed loci with low coverage (DP, total number of reads across all samples, <6000). On average, ~70 reads per sample were obtained for each remaining locus. Additionally, for those loci with a low proportion of missing data in one of the parental species (md < 0.05) but with a very high proportion in the other parental species (md > 0.90), a null allele was assigned to such missing data and to one of the alleles in homozygous individuals in the latter species; this was considered the scenario I (ScnI). These loci (hereinafter referred to as imputed loci) can correspond to alterations in one or both restriction sites (including methylation), yielding no scorable fragments in the sequencing phase, or to larger indels in the genome of one of the species; in any case, they can be highly informative. Nevertheless, in order to avoid an excessive impact of imputed loci, we conceived a second scenario (ScnII), in which we kept only one imputed locus per gene or intergenic fragment of the *Q. suber* genome (Ramos et al., 2018). The location of a locus within an intergenic fragment was assessed considering a proximity criterion using a 10 kb sliding window. This way, ScnII kept approximately 80% of the loci imputed under ScnI. In ScnIII we removed all the imputed alleles, and considered them as missing data. Finally, in the most restrictive scenario, we discarded all the loci with a proportion of missing data >90% in either of the adult *Q. suber* or *Q. ilex* populations (ScnIV). Later on, we also corrected the genotypes of the progenies according to each imputation scenario, and considered as missing data those genotypes incompatible with that of the corresponding mother tree.

**Figure 4:**
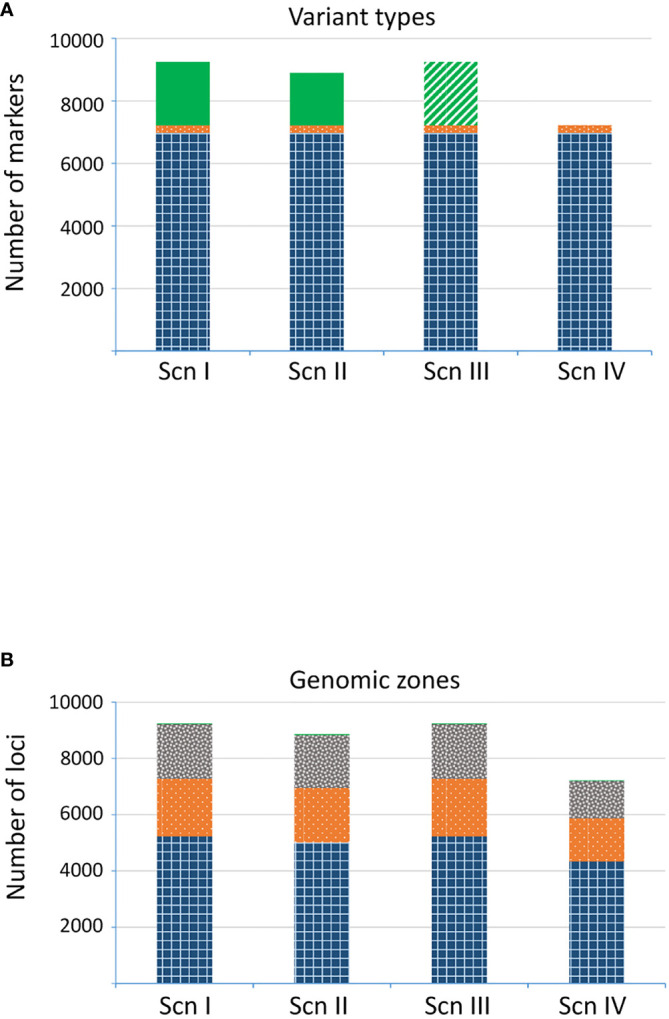
Number of loci detected for each scenario. **(A)** Variant types: SNP (gridded blue), indel (dotted orange), and imputed loci (plain green; treated as missing data in ScnIII). **(B)** Genomic region: known exonic region (gridded blue), known intronic region (dotted orange), intergenic region (grey confetti), not assigned region (plain green).

The number of final recovered loci varied depending on the scenario (**Figure 4A**). For ScnI and ScnIII we obtained up to 9,251 loci, with 2,026 (21.9%) imputed ones in ScnI. Under ScnII we considered 8,901 loci, with 18.8% of them imputed. The more restrictive ScnIV kept 7,225 unimputed loci. The annotation to the *Q. suber* genome allowed precise location of many of these loci in specific genes of known function (Ramos et al., 2018) and intergenic regions (**Figure 4B**). Loci fromScnI, ScnII and ScnIII were located in 3,396 fragments, of which 2,566 were genic and 811 intergenic. For Scn IV the number of fragments dropped to 1,829, of which 1,540 (84.2%) corresponded to genic regions and 279 (15.3%) to intergenic ones. In all the scenarios, loci corresponding to genic regions were mostly exonic c. 72%), although a significant percentage of loci (c. 28%) occurred in introns (**Figure 4B**). The remaining loci (0.3%) were located in 29 fragments that could not be mapped to the Q. suber genome assembly. Databases of markers are available at Zenodo Repository (López de Heredia et al., 2020).

### Distribution of Markers Across the Genome

**Figure 5:**
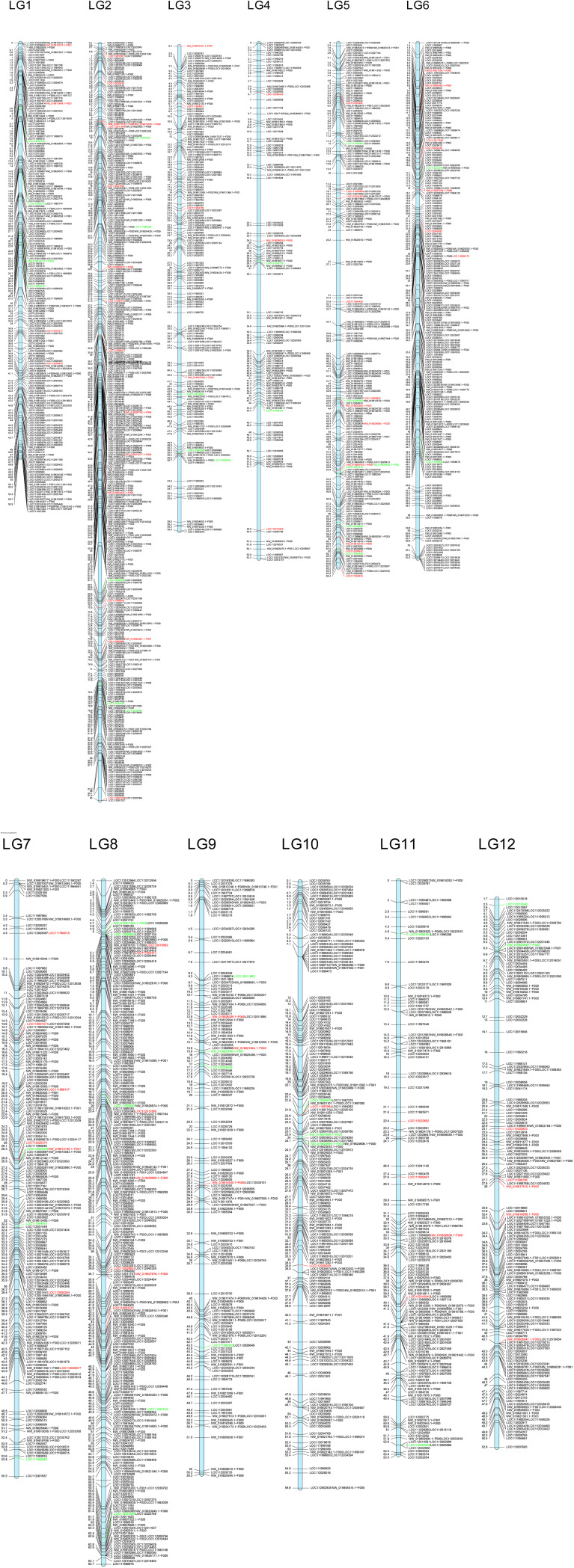
Putative location in the *Q. robur* linkage groups of the genomic fragments including markers from ScnI. Location of markers with allelic frequencies in the adult hybrids very similar to one of the parental species is highlighted in red (*Q. ilex*) or in green (*Q. suber*).

In order to evaluate their distribution across the genome, and provided there is no information regarding chromosomal distribution of *Q. suber* nor *Q. ilex* genomes, we checked the homology of these loci on the *Q. robur* genome, for which 12 linkage groups (corresponding to the 12 chromosomes) have been identified (Bodénès et al., 2012; Bodénès et al., 2016). A total of 8,774 loci were successfully mapped against *Q. robur* genome; of these, 8,004 showed homology with loci included in the 12 linkage groups. These loci belong to 2,932 genomic fragments: 2,264 genic and 668 intergenic. We found a rather even distribution of these loci among the 12 linkage groups, with an average distribution of almost 670 loci per linkage group, approximately 10.55 loci/Mb (**Figure 5**). Assuming a high synteny among species of the genus (Bodénès et al., 2012; Konar et al., 2017), these results suggest an unbiased distribution of our loci across *Q. suber* and *Q. ilex* genomes.

### Introgression Levels

**Figure 6:**
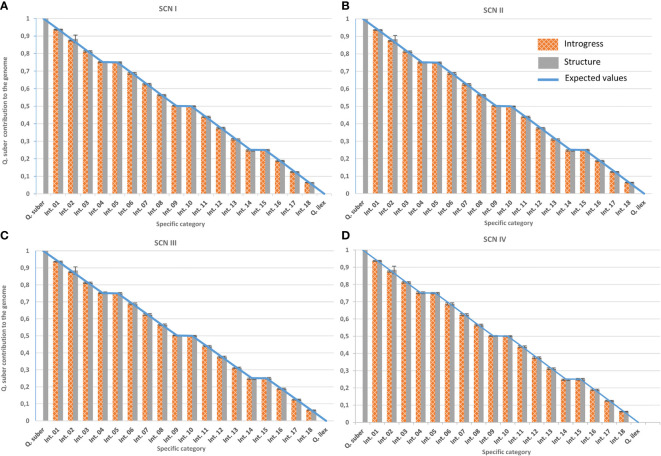
Performance of classification tools under the different imputation scenarios. Genomic contribution of *Q. suber* is estimated using Structure’s qs (plain grey) and Introgress hybrid index (gridded orange) on virtual individuals generated with SimHyb (pure species and 18 intermediate categories). Expected values are represented by a blue line. Standard deviations are also indicated. **(A)** Imputation scenario I; **(B)** imputation scenario II; **(C)** imputation scenario III; **(D)** imputation scenario IV.

The Bayesian approach implemented in Structure v. 2.3.4 (Pritchard et al., 2000), as well as the hybrid index provided by the introgress R-package (Gompert and Buerkle, 2010), were then used to classify individuals according to their introgression levels. Firstly, and following the procedure established by Burgarella et al. (2009), accuracy of the classification was evaluated using virtual individuals of known pedigree created with SimHyb (Soto et al., 2018). ScnIII yielded the same results as ScnIV, due to the distribution of missing data among species and the way both programs consider them. Therefore, ScnIII was discarded for further analysis of real individuals (**Figure 6**).

**Figure 7:**
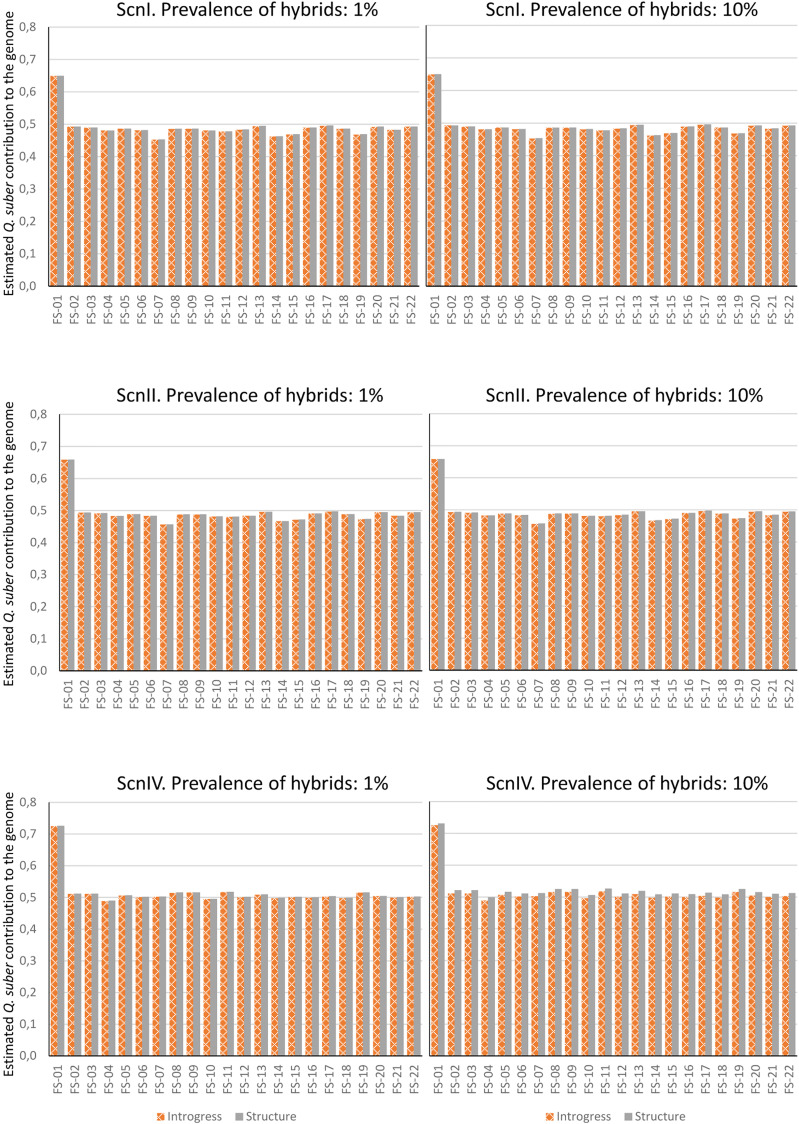
Contribution of Q. suber to the genome of adult hybrids under each scenario, estimated by means of STRUCTURE's qs and Hybrid Index of INTROGRESS, with 1% and 10% of hybrids.

Once accuracy and reliability of classification tools had been checked, introgression levels of real hybrid adults were assessed. Estimation was performed considering 1% and 10% of hybrids in the analyzed population. Introgress and Structure yielded similar results in each situation, and very small differences were detected between both hybrid prevalence situations. Under the four imputation scenarios, estimations for adult individuals were roughly compatible with F1 hybrids (except for FS-01, which could be classified as a backcross with *Q. suber*) (**Figure 7**). As for the hybrid progenies, a large variability of introgression levels was detected, although we scored, on average, a higher contribution of *Q. suber* to the genome of the seedlings for all the hybrid families, compared to their mother trees (**Table 2**).

**Table 2:** Structure’s q_s_ and Introgress hybrid index estimates for the progenies of the open-pollinated hybrid families under each scenario.

Mother tree	Scn I			Scn II			Scn IV		
	q_s_	Max	min	q_s_	Max	min	q_s_	Max	min
FS08	0.633 (0.049)	0.714	0.543	0.644 (0.051)	0.726	0.548	0.731 (0.067)	0.821	0.606
FS14	0.648 (0.096)	0.776	0.322	0.658 (0.100)	0.791	0.319	0.731 (0.125)	0.904	0.316
FS16	0.626 (0.058)	0.713	0.317	0.634 (0.061)	0.726	0.313	0.700 (0.075)	0.818	0.311
FS17	0.655 (0.073)	0.758	0.361	0.665 (0.077)	0.773	0.358	0.738 (0.096)	0.878	0.365
FS18	0.662 (0.035)	0.718	0.596	0.672 (0.037)	0.731	0.602	0.748 (0.048)	0.824	0.659
FS19	0.587 (0.152)	0.769	0.155	0.595 (0.157)	0.784	0.149	0.657 (0.190)	0.893	0.114
FS20	0.663 (0.058)	0.773	0.521	0.674 (0.061)	0.789	0.524	0.752 (0.079)	0.901	0.561
FS21	0.625 (0.013)	0.640	0.607	0.633 (0.014)	0.648	0.614	0.701 (0.019)	0.722	0.677
FS22	0.633 (0.056)	0.721	0.359	0.643 (0.058)	0.736	0.373	0.718 (0.068)	0.835	0.487

Mean, standard deviation (in brackets), maximum and minimum values per family are provided.

Following López de Heredia et al. (2018a), we compared Structure estimates of the *Q. suber* contribution to the genome of each hybrid seedling to that of its mother tree (q_soffspring_/q_smother_). Values higher than 1 point to likely fertilizations by *Q. suber*, while values below 1 suggest fertilization by *Q. ilex*. This comparison suggests that hybrid mother trees can be fertilized by both *Q. ilex* and Q*. suber* pollen, although more frequently by the latter species, at least in the studied season (**Table 3**).

**Table 3:** Ratio between Structure’s q_s_ of the offspring and q_s_ of their mothers under each scenario.

Mother tree	Scn I			Scn II			Scn IV		
	q_s_o/q_s_m	Max	Min	q_s_o/q_s_m	Max	Min	q_s_o/q_s_m	Max	Min
FS08	1.303	1.469	1.117	1.316	1.485	1.121	1.392	1.564	1.154
FS14	1.400	1.676	0.695	1.405	1.690	0.682	1.438	1.780	0.622
FS16	1.277	1.455	0.647	1.290	1.479	0.637	1.375	1.607	0.611
FS17	1.321	1.528	0.728	1.335	1.552	0.719	1.439	1.712	0.712
FS18	1.359	1.474	1.224	1.374	1.495	1.231	1.472	1.622	1.297
FS19	1.252	1.640	0.330	1.254	1.654	0.314	1.252	1.701	0.217
FS20	1.346	1.568	1.057	1.358	1.591	1.056	1.459	1.750	1.089
FS21	1.290	1.322	1.254	1.304	1.336	1.266	1.375	1.416	1.327
FS22	1.284	1.462	0.728	1.298	1.487	0.754	1.403	1.631	0.951

Mean, maximum and minimum values per hybrid family are provided. Mean for the open-pollinated hybrid families by scenario. Values >1 point to likely fertilizations by Q. suber, while values <1 suggest fertilization by Q. ilex (López de Heredia et al., 2018a).

## Discussion

Recent advances in NGS methodologies along with the release of reference genome assemblies have opened up new perspectives in the study of open pollinated plant hybridizing systems, such as the *Quercus* syngameon (Kremer and Hipp, 2020). In this work, we have used ddRADseq methodology and have developed *ad hoc* bioinformatic pipelines to identify genomic variants that may shed light in the study of ongoing hybridization and genomic boundaries between *Q. suber* and *Q. ilex.* This insight may complement the current view derived from other presumably more introgressed *Quercus* complexes, such as the intensively studied European white oaks.

Most hybridization studies in woody plant species have been conducted to assess ancient introgression from a phylogenetic perspective (McVay et al., 2017) or ongoing introgression by sampling individuals exploring ample hybrid zones (Lepais et al., 2009; Zeng et al., 2011). In this work, we have adopted a local scale approach, aiming to block the impact of environmental or IBD related variation in candidate loci inference. The sampling scheme of adults and progenies of both parental species and hybrids used here has revealed powerful to identify candidate gene markers for the study of ongoing hybridization in Mediterranean sclerophyllous oaks.

### Candidate Marker Loci Identification

Obtaining candidate loci in reduced genome representation studies (ddRADseq), particularly if they are aimed to perform individual classification of hybrids, requires careful experimental design to ensure sufficient coverage of the genome of the focal species and rigorous filtering of variants in order to minimize the potential sources of error in marker identification (O’Leary et al., 2018). In the present work, an experimental design derived from a pilot study (Guillardín-Calvo et al., 2019), together with an *ad hoc* bioinformatic pipeline has enabled the identification of a significant number of genomic markers to analyze ongoing hybridization between *Q. ilex* and *Q. suber*.

We employed an enzyme combination (PstI/MspI) that prioritizes the representation of loci within or in the proximity of genic regions that could be relevant for the study of introgression patterns, enriching for hypomethylated gene space and reducing the number of fragments from highly repetitive genomic regions (Emberton et al., 2005; Nelson et al., 2008; Poland and Rife, 2012). Moreover, a combination of methylation-sensitive rare- and common-cutting enzymes, such as PstI/MspI, has proven to show greater uniformity of read depth across loci providing high quality genotype information in plants (Pootakham et al., 2016). We have also optimized ddRADseq protocols by performing a size selection step to ensure the collection of robust polymorphic loci at sufficient depth. Actually, genome mapping and variant calling using *Q. suber* genome assembly as a reference have confirmed that most candidate polymorphic markers (*c*. 80%) correspond to genic regions, more than 55% of loci are located in exons and *c*. 22% in introns. Approximately 20% of loci were located in intergenic regions, and, comparatively few candidate loci (0.3%) were obtained from the pseudogenome mapping. However, we cannot discard that some of these regions could contain genes or regulatory regions in our focal species and further re-sequencing experimental work will be necessary to confirm this point.

To gain robustness in candidate marker inference, we have applied a filtering-imputation procedure to yield four different scenarios. Using restrictive filteringcriteria (ScnIV), we have obtained 7,225markers that correspond to 1,540 genic fragments of known function, 279 intergenic fragments and 10 fragments that could not be assigned to *Q. suber* genome assembly. Nevertheless, strict application of these criteria could have discarded informative loci, for instance, those present in the genome of one of the parental species but absent in the other. To avoid missing this information, we designed an imputation procedure, giving rise to what we have called scenarios I and II. This way we identified up to 2,026 additional loci, with imputed null alleles, under ScnI. These loci, which could be highly informative for introgression studies, belonged to 1,264 genic fragments of known function, 584 intergenic fragments and 9 fragments that could not be assigned to *Q. suber* genome assembly. It is noteworthy that many of these null alleles were imputed to *Q. ilex*.

The markers identified in this work provide a well-distributed coverage of the whole genome, assuming synteny among genomes of the genus (Bodénès et al., 2012; Konar et al., 2017), since their orthologs appear distributed throughout the 12 linkage groups described for *Q. robur* (Plomion et al., 2018) (**Figure 5**). Furthermore, we have investigated the distribution of the most discriminating genomic regions between *Q. suber* and *Q. ilex*. Thus, in addition to the 2,026 imputed loci, up to 2,830 non-imputed ones show very different patterns in both species, with frequencies of the most common allele 0.9 in one of the species and 0.2 in the other one. Regarding the hybrids, under ScnI 190 loci (167 imputed) show allelic frequencies in the hybrids quite similar to those of *Q. ilex* and very different from *Q. suber*, while 83 loci (17 imputed) show frequencies in the hybrids very similar to *Q. suber* and different from *Q. ilex*. These loci, presumably linked to introgression directionality and genomic barriers between *Q. suber* and *Q. ilex*, are scattered throughout the 12 linkage groups (**Figure 5**). This feature seems to be consistent with previous results for *Q. robur* and *Q. petraea* based on QTL (Saintagne et al., 2004), suggesting that species boundaries are underpinned by a large number of small genomic regions, rather than on few large blocks.

### Individual Introgression Levels

Most adult hybrids could be classified as F1 hybrids under all the imputation scenarios considered. Inclusion of imputed loci does not entail a significant difference in the estimation of the contribution of parental species to the genome of hybrid individuals. For the adult hybrids, only slightly lower values of *Q. suber* contribution are obtained under ScnI and ScnII. Different results are observed for the hybrid progenies. Individuals with higher estimated *Q. suber* contributions under ScnIV show lower values when imputed loci are considered, while the opposite is observed for individuals with lower estimations. Since most of the null alleles are imputed to *Q. ilex*, this latter result must be due to a higher proportion of non-imputed, “suber” alleles in heterozygosity in these loci in these individuals. Taking into account PstI/MspI sensitivity to methylation, hybridization-mediated alteration of epigenetic characters could also contribute to these results. This way, methylated epialleles in the restriction sites, which would yield no scorable reads and, therefore, would have been imputed with a null allele, could have turned out to be unmethylated and therefore scorable in hybrids, or vice-versa. This could be the case at least of the 184 markers for which very high frequencies of the imputed allele are recorded in adult hybrids ( 0.75), no matter their global classification as F1 hybrids. Such an alteration of epigenetic characters has been reported, for instance, in *Spartina* (Salmon et al., 2005) or in *Solanum* (Marfil et al., 2006). On the contrary, Radosavljevic et al. (2019) obtained similar estimations of introgression levels using markers sensitive or insensitive to methylation in *Salvia officinalis* and *S. fruticosa* hybrids. Further research will be needed to disentangle the effects of *Q. ilex* and *Q. suber* hybridization in epigenetic patterns.

On the other hand, estimated contribution of *Q. suber* increases in the genome of hybrid offspring compared with their mother trees, for all the families and in all the scenarios considered. These results suggest a preferential fertilization of hybrid trees by *Q. suber* pollen. Different factors, including phenology can account for this feature. In fact, earlier flowering of *Q. ilex* (Gómez-Casero et al., 2007) favors that initial hybridization takes place with *Q. ilex* acting as mother tree; additionally, pre-zygotic incompatibilities for *Q. ilex* pollen in *Q. suber* pistil have also been reported (Boavida et al., 2001). Consistently, all the 22 adult hybrid individuals included in this study carry *ilex* chloroplasts. Actually, *ilex* chloroplasts have been found in all hybrid trees analyzed to date (Lumaret et al., 2002; Burgarella et al., 2009; López de Heredia et al., 2018a). Thus, hybrid mother trees could be preferentially fertilized by *Q. suber.* The only study performed to date, to our knowledge, reports a similar reproductive phenology for hybrid trees and *Q. suber* (Perea Garcia-Calvo, 2006), so that backcrossing with this species would be more likely. Additionally, pre- and post-zygotic incompatibilities or a better performance of *Q. suber* pollen tubes in the hybrid pistil could also account for such a preferential fertilization. In spite of this, López de Heredia et al. (2018a) reported that hybrids can be effectively pollinated both by *Q. suber* and by *Q. ilex* and that backcrossing directionality is largely driven by pollen availability. Consistently, and notwithstanding the preferential fertilization by *Q. suber*, in the present work we also report some hybrid seedlings presumably derived from *Q. ilex* pollen in most families. This result is also in accordance with the detection of putatively introgressed holm oaks reported by Burgarella et al. (2009). Moreover, our analyses yield intermediate contribution of parental species to most hybrid seedlings (according to both Structure and Introgress). This feature in hybrid mother trees contrasts with Cannon and Scher (2017) hypothesis for hybrid pollen and should not be disregarded. Actually, it may be more likely that hybrid trees scattered in stands of parental species reproduce acting as mother trees, pollinated by parental species, than as pollen donors. In that case, there is no reason to suppose any advantage for pure parental gametes in a diploid, hybrid stigma or for “pure” or “almost pure” zygotes and seed development in the hybrid sporophyte (mother tree).

The larger contribution from *Q. suber* to hybrid progenies’ genomes compared to the adults could be due to greater availability of *Q. suber* pollen in the only campaign analyzed in the present study. However, a selective advantage for individuals more similar to *Q. ilex*, from the seedling to the adult stage cannot be discarded either. Such a selection would not be unexpected. Fertilization of hybrids can give rise to a huge amount of genomic combinations. Large part of them can be incompatible since the first developmental stages, hampering the formation of embryos. Later on, other combinations can also be deleterious, as confirmed by the low germination rate of hybrid acorns in this study (56.92% in hybrids, compared to 89.24% in *Q. suber* and up to 92.95% in *Q. ilex*), consistent with previous studies in other areas (López de Heredia et al., 2018a). Although many morphological anomalies already described in *Q. suber x ilex* hybrid seedlings (López de Heredia et al., 2017; López de Heredia et al., 2018b) were present in part of the seedlings, we have scored low mortality in nursery conditions over three years since seedlings emergence. Nevertheless, selection against certain genomic combinations—late-acting genetic incompatibilities—during the juvenile phases of hybrid individuals with major *Q. suber* ancestry could also account for the higher contribution of *Q. ilex* to the genome of surviving hybrids. In addition, extrinsic selection during pre-adult stages, mitigated under nursery conditions, cannot be discarded.

## Conclusion and Future Prospects

Our work reports a case study of hybridization and introgression in two non-model forest tree species, *Q. suber* and *Q. ilex*, using genome-wide NGS techniques, and provides a pipeline and scripts for this kind of studies. Out of the 9,251 marker loci identified in this study, 4,856 are highly discriminant between both species, and 2,026 of these are apparently absent in one of the species (*Q. ilex* in most cases). This can be due to alterations in restriction enzyme target sites or to real indels. Interestingly, for 9.1%of them adult hybrids show patterns quite similar to one of the parental species (8.3% to *Q. ilex*, while only 0.8% to *Q. suber*), suggesting selection of those alleles in backcrosses or hybridization-mediated alterations of the methylation patterns. In any case, these loci deserve further attention, since they could be linked to viability of hybrid individuals and/or to selective advantages.

### Future Prospects

Most discriminant loci and their adjacent regions, as well as those putatively involved in selection of hybrids, should be analyzed both in hybrid individuals and in parental species. These genomic regions are good candidates to be involved in genome permeability, interspecific barriers and, eventually, in adaptive introgression. Further characterization of these loci, as well as of those with different allele frequencies in hybrid adults and seedlings could provide insight in species boundaries and on adaptive introgression between *Q. suber* and *Q. ilex.*

## Data Availability Statement

The datasets presented in this study can be found in online repositories. The names of the repository/repositories and accession number(s) can be found below: https://www.ncbi.nlm.nih.gov/genbank/, LR797857-LR797862 https://www.ncbi.nlm.nih.gov/, PRJNA628590.

## Author Contributions

AS conceived the idea and drafted the manuscript. AS and ULH designed the experiments and collected the plant material. LG-C handled the plant material. MS performed the cpDNA analysis. FM-M, PGG, ULH, and AS performed the simulations and bioinformatics analysis. All the authors contributed to the article and approved the submitted version.

## Funding

This work was funded by the projects AGL2015-67495-C2-2-R (Spanish Ministry of Economy and Competitiveness) and PID2019-110330GB-C22 (Spanish Ministry of Science and Innovation).

## Conflict of Interest

The authors declare that the research was conducted in the absence of any commercial or financial relationships that could be construed as a potential conflict of interest.

The reviewer JM declared a past co-authorship with one of the authors MS to the handling Editor.
