# Corrigendum: ddRAD Sequencing-Based Identification of Genomic Boundaries and Permeability in *Quercus ilex* and *Q. suber* Hybrids

**DOI:** 10.3389/fpls.2021.786695

**Published:** 2022-01-10

**Authors:** Unai López de Heredia, Fernando Mora-Márquez, Pablo G. Goicoechea, Laura Guillardín-Calvo, Marco C. Simeone, Álvaro Soto

**Affiliations:** ^1^G.I. Genética, Fisiología e Historia Forestal, Dpto. Sistemas y Recursos Naturales, ETSI Montes, Forestal y del Medio Natural, Universidad Politécnica de Madrid, Madrid, Spain; ^2^Department of Forestry, NEIKER-BRA, Vitoria-Gasteiz, Spain; ^3^Dipartimento di Scienze Agrarie e Forestali (DAFNE), Università degli Studi della Tuscia, Viterbo, Italy

**Keywords:** ddRADSeq, *Quercus*, hybridization, introgression, genomic boundaries, SNPs

The article originally published contained errors caused by mistakes made in the automated variant calling process, which led to the misidentification of several polymorphic loci. This affected mainly to the exact number of markers of different classes, reported in several sections of the text. The details of the affected parts of the article are specified here.

**1. In the** ***Abstract***

The sentence “We have identified up to 9,435 markers across the genome and have estimated individual introgression levels in adults and seedlings.” should read “We have identified up to 9,251 markers across the genome and have estimated individual introgression levels in adults and seedlings.”

Furthermore, the sentences “A noticeable proportion of the markers (26%) showed allelic frequencies in adult hybrids very similar to one of the parental species, and very different from the other; a finding that seems relevant for understanding the hybridization process and the occurrence of adaptive introgression. Candidate marker databases developed in this study constitute a valuable resource to design large scale re-sequencing experiments in Mediterranean sclerophyllous oak species and could provide insight in species boundaries and on adaptive introgression between *Q. suber* and *Q. ilex.”* should read “In adult hybrids 273 markers (3%) showed allelic frequencies very similar to one of the parental species, and very different from the other; these loci could be relevant for understanding the hybridization process and the occurrence of adaptive introgression. Candidate marker databases developed in this study constitute a valuable resource to design large scale re-sequencing experiments in Mediterranean sclerophyllous oak species and could provide insight into species boundaries and adaptive introgression between *Q. suber* and *Q. ilex*.”

**2. In the** ***Results*****, Subsection** ***Read Alignment, Variant Filtering and Imputation***

In the second paragraph, the sentences “After individual variant calling, the number of variants ranged between 14,666 and 539,229 for the genome and between 217 and 71,716 for the pseudogenome alignments. The final concatenated-merged variant calling file had 17,289,128 variants, of which >99.5% were SNPs and <8% multi-allelic sites.” should read “After individual variant calling, the number of variants ranged between 14,593 and 524,458 for the genome and between 212 and 66,680 for the pseudogenome alignments. The final concatenated-merged variant calling file had 16,234,798 variants, of which 97.4 % were SNPs and <5% multi-allelic sites.”

In the third paragraph, the sentence “This way, ScnII kept approximately 2/3 of the loci imputed under ScnI.” should read “This way, ScnII kept approximately 80% of the loci imputed under ScnI.”

In the fourth paragraph, the sentences “The number of final recovered loci varied depending on the scenario (**Figure 4A**). For ScnI and ScnIII we obtained up to 9,435 loci, with 36.6% of imputed ones in ScnI. Under ScnII we considered 8,175 loci, with 26.6% of them imputed. The more restrictive ScnIV kept 6,001 unimputed loci.” should read “The number of final recovered loci varied depending on the scenario (**Figure 4A**). For ScnI and ScnIII we obtained up to 9,251 loci, with 2,026 (21.9%) imputed ones in ScnI. Under ScnII we considered 8,901 loci, with 18.8% of them imputed. The more restrictive ScnIV kept 7,225 unimputed loci.”

Furthermore, in the fourth paragraph, the sentences “Loci from ScnI/ScnIII were located in 3,156 fragments, of which 2,406 were genic and 750 intergenic. Under ScnII only two intergenic fragments were completely discarded, resulting in a total of 3,154 identified fragments. For ScnIV the number of fragments dropped to 2,166, of which 1,577 (72.8%) corresponded to genic regions and 589 (27.2%) to intergenic ones. In all the scenarios, loci corresponding to genic regions were mostly exonic (>50%), although a significant percentage of loci (*c*. 20%) occurred in introns (**Figure 4B**). The remaining loci (3–4%) were located in 107 fragments that could not be mapped to the *Q. suber* genome assembly.” should read “Loci from ScnI, ScnII and ScnIII were located in 3,396 fragments, of which 2,566 were genic and 811 intergenic. For Scn IV the number of fragments dropped to 1,829, of which 1,540 (84.2%) corresponded to genic regions and 279 (15.3%) to intergenic ones. In all the scenarios, loci corresponding to genic regions were mostly exonic *c*. 72%), although a significant percentage of loci (*c*. 28%) occurred in introns (**Figure 4B**). The remaining loci (0.3%) were located in 29 fragments that could not be mapped to the *Q. suber* genome assembly.”

**3. In the** ***Results*****, Subsection** ***Distribution of Markers Across the Genome***

The sentences “A total of 8,210 loci were successfully mapped against *Q. robur* genome; of these, 7,559 showed homology with loci included in the 12 linkage groups. These loci belong to 2,764 genomic fragments: 2,110 genic, 646 intergenic, and 8 fragments not found in the *Q. suber* genome. We found a rather even distribution of these loci among the 12 linkage groups, with an average distribution of more than 600 loci per linkage group, approximately 10.55 loci/Mb (**Figure 5**).” should read “A total of 8,774 loci were successfully mapped against *Q. robur* genome; of these, 8,004 showed homology with loci included in the 12 linkage groups. These loci belong to 2,932 genomic fragments: 2,264 genic and 668 intergenic. We found a rather even distribution of these loci among the 12 linkage groups, with an average distribution of almost 670 loci per linkage group, approximately 10.55 loci/Mb (**Figure 5**).”

**4. In the** ***Results*****, Subsection** ***Introgression Levels***

In the first paragraph, the sentences “ScnIV provided a fairly accurate classification of virtual hybrid individuals. ScnII, and, most of all, ScnI, provided even more precise classifications. On the contrary, ScnIII yielded large deviations for virtual individuals. Therefore, ScnIII was discarded for further analysis of real individuals (**Figure 6**).” should read “ScnIII yielded the same results as ScnIV, due to the distribution of missing data among species and the way both programs consider them. Therefore, ScnIII was discarded for further analysis of real individuals (**Figure 6**).”

In the second paragraph, the sentences, “Estimation was performed considering 1 and 10% of hybrids in the analyzed population. INTROGRESS and STRUCTURE yielded similar results in each situation, and very small differences were detected between both hybrid prevalence situations. On the contrary, noticeably different results were obtained for ScnI and ScnII on one hand, and ScnIV on the other. A much larger contribution of *Q. ilex* was estimated under ScnI and ScnII. Only FS-01 showed a roughly similar contribution of both parental species while the rest of hybrids could be rather classified as backcrosses with *Q. ilex*. Under ScnIV, estimations for adult individuals were roughly compatible with F1 hybrids (except for FS-01, which could be classified as a backcross with *Q. suber*) (**Figure 7**).” Should read “Estimation was performed considering 1% and 10% of hybrids in the analyzed population. Introgress and Structure yielded similar results in each situation, and very small differences were detected between both hybrid prevalence situations. Under the four imputation scenarios, estimations for adult individuals were roughly compatible with F1 hybrids (except for FS-01, which could be classified as a backcross with *Q. suber*) (**Figure 7**).”

**5. In the** ***Discussion*****, Subsection** ***Candidate Marker Loci Identification***

In the second paragraph, the sentences “Actually, genome mapping and variant calling using *Q. suber* genome assembly as a reference have confirmed that most candidate polymorphic markers (*c*. 73%) correspond to genic regions, more than 50% of loci are located in exons and *c*. 20% in introns. Approximately 25% of loci were located in intergenic regions, and, comparatively few candidate loci (*c*. 3%) were obtained from the pseudogenome mapping.” should read “Actually, genome mapping and variant calling using *Q. suber* genome assembly as a reference have confirmed that most candidate polymorphic markers (*c*. 80%) correspond to genic regions, more than 55% of loci are located in exons and *c*. 22% in introns. Approximately 20% of loci were located in intergenic regions, and, comparatively few candidate loci (0.3%) were obtained from the pseudogenome mapping.”

In the third paragraph, the sentence “Using restrictive filtering criteria (ScnIV), we have obtained 6,001 markers that correspond to 1,577 genic fragments of known function, 489 intergenic fragments, and 107 fragments that could not be assigned to *Q. suber* genome assembly.” should read “Using restrictive filtering criteria (ScnIV), we have obtained 7,225 markers that correspond to 1,540 genic fragments of known function, 279 intergenic fragments and 10 fragments that could not be assigned to *Q. suber* genome assembly.”

Furthermore, in the third paragraph, the sentences “This way we identified up to 3,434 additional loci, with imputed null alleles, under ScnI. These loci, which could be highly informative for introgression studies, belonged to 2,406 genic fragments of known function, 750 intergenic fragments, and 107 fragments that could not be assigned to *Q. suber* genome assembly. It is noteworthy that many of these null alleles were imputed to *Q. suber*. Given the large number of imputed loci and their asymmetric distribution between both species, we prepared an additional filtering of imputed loci (ScnIII), considering as missing data the imputed alleles from ScnI. However, estimations of the introgression levels for simulated individuals showed a poor accuracy under ScnIII; therefore, it was discarded in further analysis.” should read “This way we identified up to 2,026 additional loci, with imputed null alleles, under ScnI. These loci, which could be highly informative for introgression studies, belonged to 1,264 genic fragments of known function, 584 intergenic fragments and 9 fragments that could not be assigned to *Q. suber* genome assembly. It is noteworthy that many of these null alleles were imputed to *Q. ilex*.”

In the fourth paragraph, the sentence “Thus, 2,457 loci under ScnI show allelic frequencies in the hybrids quite similar to those of *Q. ilex* and very different from *Q. suber*, while just 34 loci show frequencies in the hybrids very similar to *Q. suber* and different from *Q. ilex*.” should read “Thus, in addition to the 2,026 imputed loci, up to 2,830 non-imputed ones show very different patterns in both species, with frequencies of the most common allele ≥0.9 in one of the species and ≤ 0.2 in the other one. Regarding the hybrids, under ScnI 190 loci (167 imputed) show allelic frequencies in the hybrids quite similar to those of *Q. ilex* and very different from *Q. suber*, while 83 loci (17 imputed) show frequencies in the hybrids very similar to *Q. suber* and different from *Q. ilex*.”

**6. In the** ***Discussion*****, Subsection** ***Individual Introgression Levels***

In the first paragraph the sentences “As pointed out above, the estimations under ScnI on one side and under ScnIV on the other constitute the limits between which real introgression levels probably lie. Under ScnIV, which considers up to 6,001 markers, most adult hybrids could be classified as F1 hybrids. On the contrary, it is noteworthy that inclusion of imputable loci in the analysis (ScnI and, to a lesser extent, ScnII), yields a higher contribution of *Q. ilex* to adult hybrid genomes compared to ScnIV. Since most of the null alleles are imputed to *Q. suber*, this result must be due to a higher proportion of non-imputed, “*ilex”* alleles in heterozygosity in these loci in adult hybrids. Taking into account PstI/MspI sensitivity to methylation, hybridization-mediated alteration of epigenetic characters could also contribute to the apparent higher contribution of *Q. ilex* to the genome of hybrid individuals. This way, methylated epialleles in the restriction sites in *Q. suber*, which would yield no scorable reads and, therefore, would have been imputed with a null allele, could have turned out to be unmethylated and therefore scorable in hybrids, yielding an apparent higher contribution of *Q. ilex* even to F1 hybrids.” should read “Most adult hybrids could be classified as F1 hybrids under all the imputation scenarios considered. Inclusion of imputed loci does not entail a significant difference in the estimation of the contribution of parental species to the genome of hybrid individuals. For the adult hybrids, only slightly lower values of *Q. suber* contribution are obtained under ScnI and ScnII. Different results are observed for the hybrid progenies. Individuals with higher estimated *Q. suber* contributions under ScnIV show lower values when imputed loci are considered, while the opposite is observed for individuals with lower estimations. Since most of the null alleles are imputed to *Q. ilex*, this latter result must be due to a higher proportion of non-imputed, “*suber”* alleles in heterozygosity in these loci in these individuals. Taking into account PstI/MspI sensitivity to methylation, hybridization-mediated alteration of epigenetic characters could also contribute to these results. This way, methylated epialleles in the restriction sites, which would yield no scorable reads and, therefore, would have been imputed with a null allele, could have turned out to be unmethylated and therefore scorable in hybrids, or vice-versa. This could be the case at least of the 184 markers for which very high frequencies of the imputed allele are recorded in adult hybrids (≥0.75), no matter their global classification as F1 hybrids.”

**7. In the Section** ***Conclusion and Future Prospect***

The first and second paragraphs “Our work reports a case study of hybridization and introgression in two non-model forest tree species, *Q. suber* and *Q. ilex*, using genome-wide NGS techniques, and provides a pipeline and scripts for this kind of studies. We have identified up to 9,435 marker loci in *Q. suber* and *Q. ilex*. Among them, allelic frequencies of 2,457 are quite similar in hybrid adult individuals and in *Q. ilex*, while only 34 are quite similar in hybrids and *Q. suber*, consistently with the estimated higher contribution of this latter species to the genome of adult hybrids.

Additionally, we have detected 3,434 highly discriminating loci for which a species-specific null allele has been imputed. In most cases, the fragment was scored in *Q. ilex* samples, and absent in *Q. suber*. This can be due to alterations in restriction enzyme target sites or to real indels. Interestingly, in many cases hybrid individuals show the presence of *Q. ilex* variants, rather than *Q. suber* variants, suggesting a selection of these alleles in backcrosses or hybridization-mediated alterations of the methylation patterns. In any case, these loci deserve further attention, since they could be linked to viability of hybrid individuals or to selective advantages.” should read (after being merged into a single paragraph). “Our work reports a case study of hybridization and introgression in two non-model forest tree species, *Q. suber* and *Q. ilex*, using genome-wide NGS techniques, and provides a pipeline and scripts for this kind of studies. Out of the 9,251 marker loci identified in this study, 4,856 are highly discriminant between both species, and 2,026 of these are apparently absent in one of the species (*Q. ilex* in most cases). This can be due to alterations in restriction enzyme target sites or to real indels. Interestingly, for 9.1% of them adult hybrids show patterns quite similar to one of the parental species (8.3% to *Q. ilex*, while only 0.8% to *Q. suber*), suggesting selection of those alleles in backcrosses or hybridization-mediated alterations of the methylation patterns. In any case, these loci deserve further attention, since they could be linked to viability of hybrid individuals and/or to selective advantages.

**8**. ***Materials and Methods*****, Subsection** ***Estimation of Introgression Levels***

In the original article, the correct website for downloading the SimHyb software was not cited. Therefore, the sentence “These individuals were simulated with SIMHYB (Soto et al., 2018), based on the allele frequencies of the adult *Q. ilex* and *Q. suber* populations.” should read “These individuals were simulated with SimHyb (Soto et al., 2018; https://github.com/GGFHF/SimHyb), based on the allele frequencies of the adult *Q. ilex* and *Q. suber* populations.”

**9. Table Errors**

In the original article, there were some mistakes in Tables 2, 3 as published. The tables corresponded to the results obtained after an incorrect variant calling procedure. The corrected Tables 2, 3 appear here.

**Table 2:** STRUCTURE's q_s_ and INTROGRESS hybrid index estimates for the progenies of the open-pollinated hybrid families under each scenario.

**Mother tree**	**Scn I**			**Scn II**			**Scn IV**		
	**q_**s**_**	**Max**	**Min**	**q_**s**_**	**Max**	**Min**	**q_**s**_**	**Max**	**Min**
FS08	0.633 (0.049)	0.714	0.543	0.644 (0.051)	0.726	0.548	0.731 (0.067)	0.821	0.606
FS14	0.648 (0.096)	0.776	0.322	0.658 (0.100)	0.791	0.319	0.731 (0.125)	0.904	0.316
FS16	0.626 (0.058)	0.713	0.317	0.634 (0.061)	0.726	0.313	0.700 (0.075)	0.818	0.311
FS17	0.655 (0.073)	0.758	0.361	0.665 (0.077)	0.773	0.358	0.738 (0.096)	0.878	0.365
FS18	0.662 (0.035)	0.718	0.596	0.672 (0.037)	0.731	0.602	0.748 (0.048)	0.824	0.659
FS19	0.587 (0.152)	0.769	0.155	0.595 (0.157)	0.784	0.149	0.657 (0.190)	0.893	0.114
FS20	0.663 (0.058)	0.773	0.521	0.674 (0.061)	0.789	0.524	0.752 (0.079)	0.901	0.561
FS21	0.625 (0.013)	0.640	0.607	0.633 (0.014)	0.648	0.614	0.701 (0.019)	0.722	0.677
FS22	0.633 (0.056)	0.721	0.359	0.643 (0.058)	0.736	0.373	0.718 (0.068)	0.835	0.487

*Mean, standard deviation (in brackets), maximum and minimum values per family are provided*.

**Table 3:** Ratio between STRUCTURE's q_s_ of the offspring and q_s_ of their mothers under each scenario.

**Mother tree**	**Scn I**			**Scn II**			**Scn IV**		
	**q_**s**_o/q_**s**_m**	**Max**	**Min**	**q_**s**_o/q_**s**_m**	**Max**	**Min**	**q_**s**_o/q_**s**_m**	**Max**	**Min**
FS08	1.303	1.469	1.117	1.316	1.485	1.121	1.392	1.564	1.154
FS14	1.400	1.676	0.695	1.405	1.690	0.682	1.438	1.780	0.622
FS16	1.277	1.455	0.647	1.290	1.479	0.637	1.375	1.607	0.611
FS17	1.321	1.528	0.728	1.335	1.552	0.719	1.439	1.712	0.712
FS18	1.359	1.474	1.224	1.374	1.495	1.231	1.472	1.622	1.297
FS19	1.252	1.640	0.330	1.254	1.654	0.314	1.252	1.701	0.217
FS20	1.346	1.568	1.057	1.358	1.591	1.056	1.459	1.750	1.089
FS21	1.290	1.322	1.254	1.304	1.336	1.266	1.375	1.416	1.327
FS22	1.284	1.462	0.728	1.298	1.487	0.754	1.403	1.631	0.951

*Mean, maximum and minimum values per hybrid family are provided. Mean for the open-pollinated hybrid families by scenario. Values > 1 point to likely fertilizations by Q. suber, while valuses <1 suggest fertilization by Q. ilex (López de Heredia et al., 2018a)*.

**10. Figure Errors**

In the original article, there were some mistakes in Figures 4–7 as published. The figures corresponded to the results obtained after an incorrect variant calling procedure. The corrected Figures 4–7 appear here. Furthermore, in the original article, there were some mistakes in the legends for Figures 4–7 as published. The figures corresponded to the results obtained after an incorrect variant calling procedure, and the legends were phrased accordingly. The correct legends appear here.

**Figure 4:**
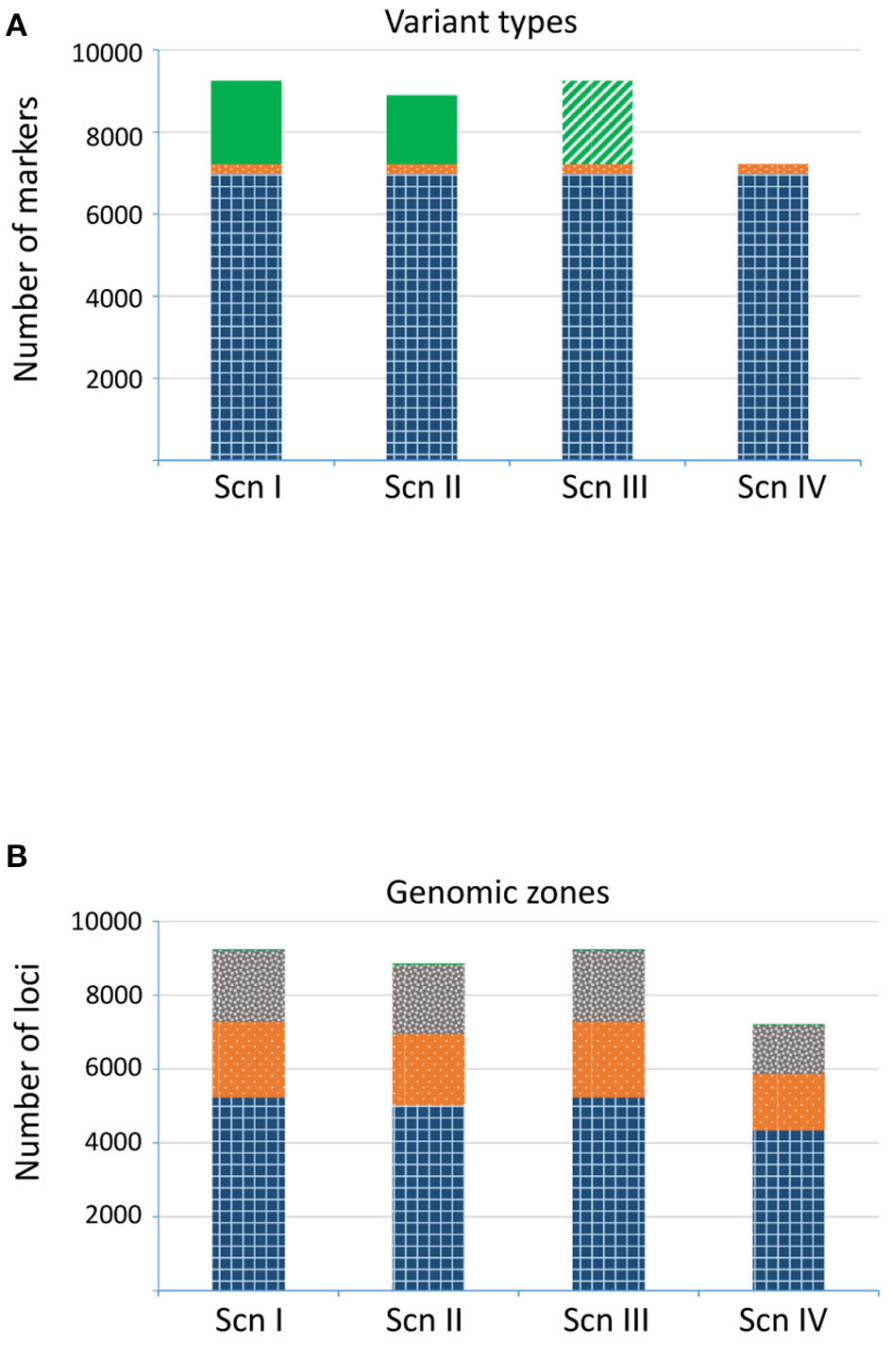
Number of loci detected for each scenario. **(A)** Variant types: SNP (gridded blue), indel (dotted orange), and imputed loci (plain green; treated as missing data in ScnIII). **(B)** Genomic region: known exonic region (gridded blue), known intronic region (dotted orange), intergenic region (gray confetti), not assigned region (plain green).

**Figure 5:**
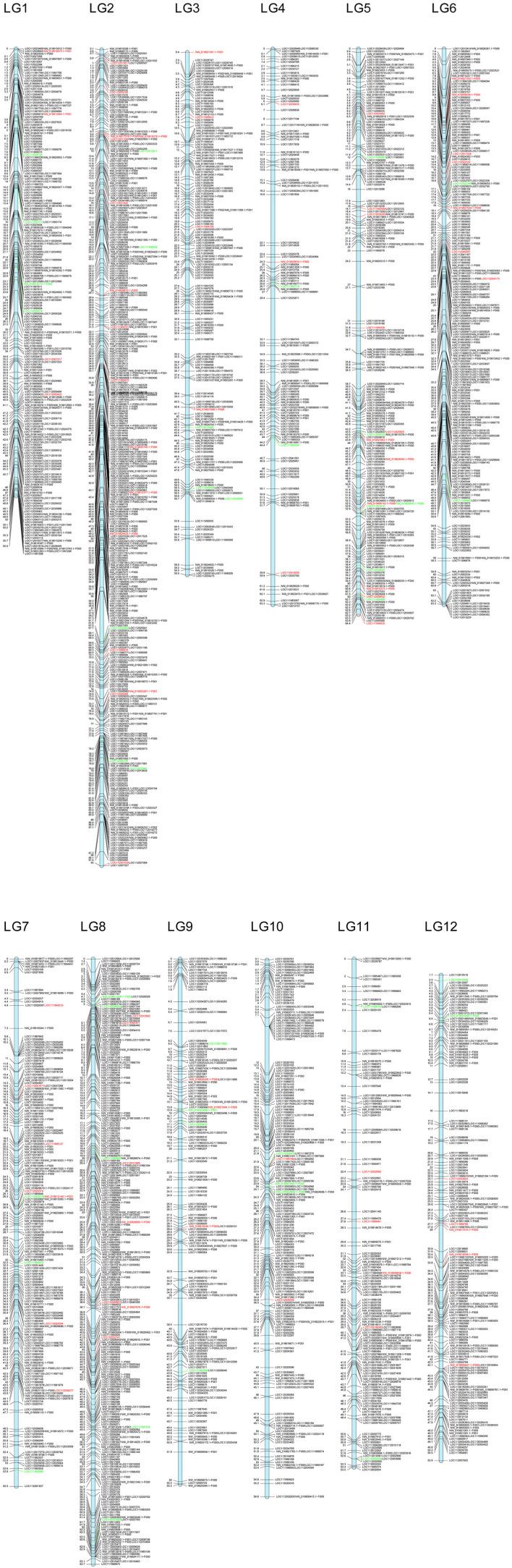
Putative location in the *Q. robur* linkage groups of the genomic fragments including markers from ScnI. Location of markers with allelic frequencies in the adult hybrids very similar to one of the parental species is highlighted in red (*Q. ilex*) or in green (*Q. suber*).

**Figure 6:**
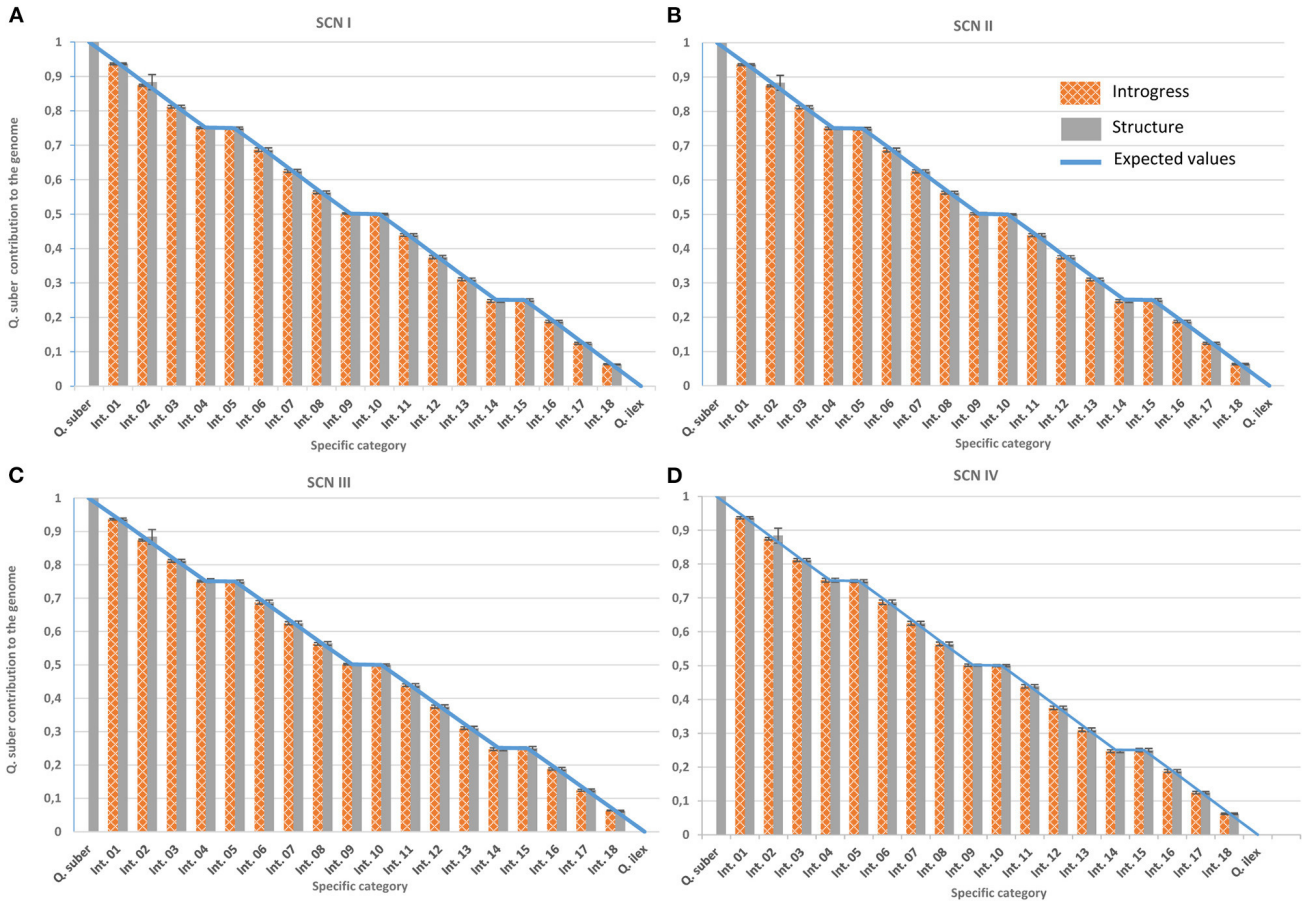
Performance of classification tools under the different imputation scenarios. Genomic contribution of *Q. suber* is estimated using Structure's q_s_ (plain grey) and Introgress hybrid index (gridded orange) on virtual individuals generated with SimHyb (pure species and 18 intermediate categories). Expected values are represented by a blue line. Standard deviations are also indicated. **(A)** imputation scenario I; **(B)** imputation scenario II; **(C)** imputation scenario III; **(D)** imputation scenario IV.

**Figure 7:**
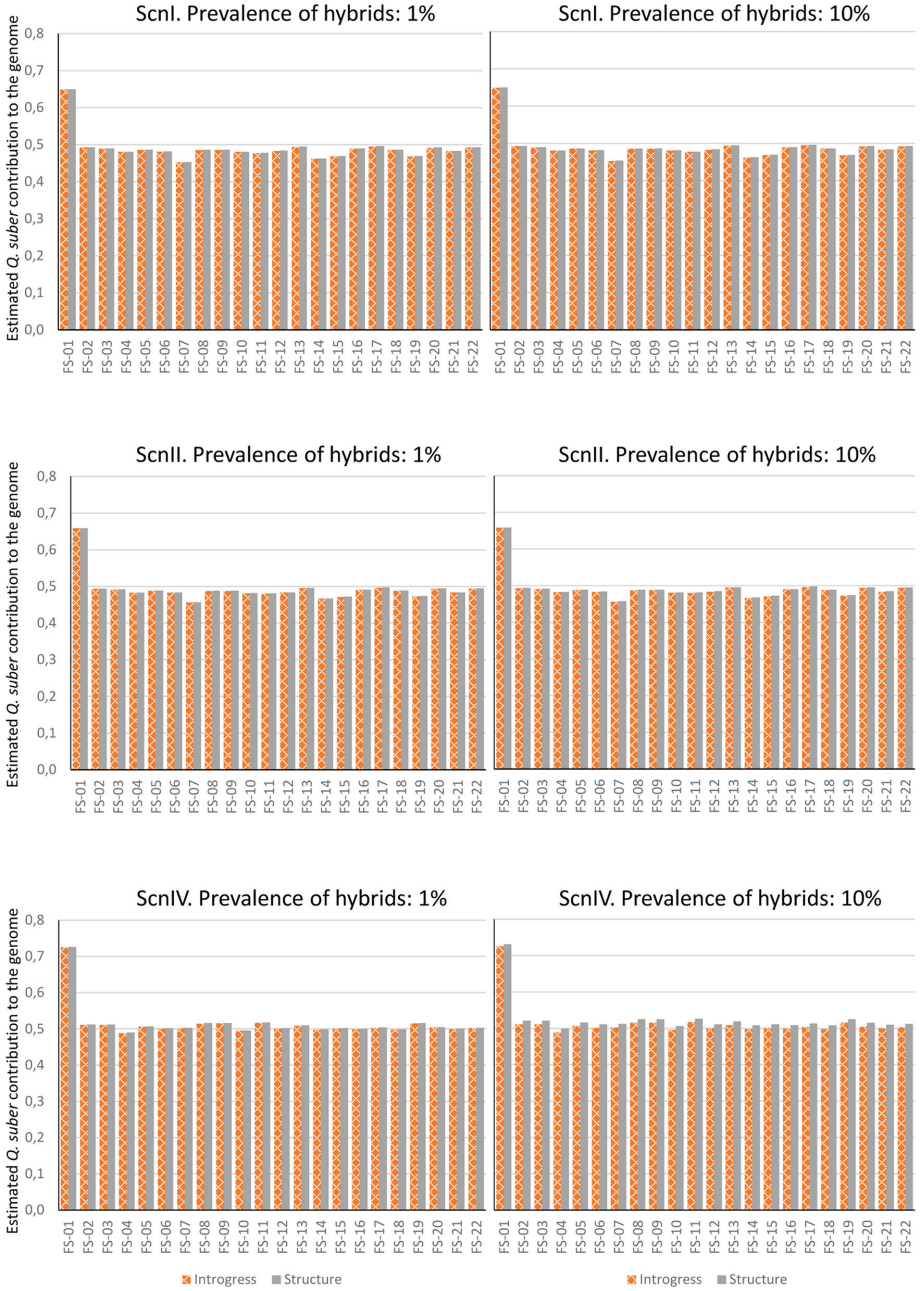
Contribution of *Q. suber* to the genome of adult hybrids under each scenario, estimated by means of Structure's qs and Hybrid Index of Introgress, with 1% and 10% of hybrids.

The authors apologize for this error and state that this does not change the scientific conclusions of the article in any way. The original article has been updated.

## Publisher's Note

All claims expressed in this article are solely those of the authors and do not necessarily represent those of their affiliated organizations, or those of the publisher, the editors and the reviewers. Any product that may be evaluated in this article, or claim that may be made by its manufacturer, is not guaranteed or endorsed by the publisher.
