# Supplementary material for: ddRAD Sequencing-Based Identification of Genomic Boundaries and Permeability in Quercus ilex and Q. suber Hybrids
Source: Front Plant Sci. 2020 Sep 4;11:564414. doi: 10.3389/fpls.2020.564414 (PMC7498617; doi:10.3389/fpls.2020.564414)
Supplement: Supplementary file 1 [file Table_1.docx]

**Supplementary List 1. Plastid (trnH-psbA) haplotype list**

**H1** = Samples EFS1, EFS2, EFS3, EFS4, EFS6, EFS8, EFS9, EFS10, EFS14, EFS16, FS14_02, FS14_06, FS08_03, FS08_11, FS16_09, FS16_25, E30, E70, E80. This haplotype is 100% identical to five GenBank sequences of *Q. ilex* (LT222181, LM652779, LM652780) and *Q. coccifera* (LT222142, LT222148) from Spain, belonging to the ‘Euro-Med’ lineage of *Quercus* sect. *Ilex* (sub-lineage V; Vitelli et al. 2017)

**H2** = Samples EFS11, EFS15, EFS17, EFS18, EFS20, EFS21, FS17_12, FS17_34, FS18_04, FS18_14, FS20_12, FS20_41, FS21_02, FS21_04, E60

**H3** = Samples EFS5, EFS7, EFS13, EFS19, EFS22, FS19_06, FS19_54, FS22_01, FS22_71. This haplotype is 100% identical to 11 GenBank sequences of *Q. ilex* (LM652784 - LM652787, LM652792, LM652820, LM652823, LT222172, LT222173, LT222194, LT222195) from Spain, France, Morocco, Algeria, Tunisia, Greece, and to three *Q. coccifera* sequences from Lybia and Spain (LT222155, LT222170, LT222141), belonging to the ‘Euro-Med’ lineage of *Quercus* sect. *Ilex* (sub-lineage V; Vitelli et al. 2017). This haplotype is also displayed by a *Q. suber* individual from Spain introgressed into *Q. ilex* (LT963529; Simeone et al. 2018)

**H4** = Samples M1_Is, M_ZLR, M1_LG, M2_LG

**H5** = Sample EFS12. This haplotype is 100% identical to a GenBank sequence of *Q. coccifera* from Portugal (HE591262), belonging to the ‘Euro-Med’ lineage of *Quercus* sect. *Ilex* (sub-lineage V; Vitelli et al. 2017)

**H6** = GenBank sequence LM652824 (*Q. ilex*, Spain), belonging to the ‘Euro-Med’ lineage of *Quercus* sect. *Ilex* (sublineage V; Vitelli et al. 2017)

**H7** = GenBank sequence LM652821 (*Q. ilex*, Morocco), belonging to the ‘Euro-Med’ lineage of *Quercus* sect. *Ilex* (sub-lineage V; Vitelli et al. 2017)

**H8** = Haplotype displayed by a *Q. suber* individual from Spain introgressed into *Q. ilex* (LT963530; Simeone et al. 2018)

**H9** = Genbank Sequences HE591299 and LT222180 (*Q. ilex*, Spain), belonging to the ‘Euro-Med’ lineage of *Quercus* sect. *Ilex* (sub-lineage V; Vitelli et al. 2017)

**H10** = Haplotype shared by *Q. ilex* from Spain, Croatia, Greece (LT222183, LT222178, LT222188), and *Q. coccifera* from Greece and Croatia (LT222140, LT222146, LT222149, LM652771, HE591300), belonging to the ‘Euro-Med’ lineage of *Quercus* sect. *Ilex* (sub-lineage V; Vitelli et al. 2017)

**H11** = Haplotype shared by several accessions of *Q. coccifera* and *Q. ilex* from Bulgary, Greece and Turkey, *Q. suber*, *Q. cerris*, *Q. trojana*, *Q. macrolepis* from Italy and Greece, belonging to the ‘Cerris-Ilex’ lineage of *Quercus* section *Ilex* (Vitelli et al. 2017). Representative sequence: LM652777

**H12** = Samples AL1, AL10, AL50, AL70. This haplotype is 100% identical to a possible ancestral ‘Cerris-Ilex’ haplotype collecting GenBank sequences of numerous species of section *Cerris* (including *Q. suber*) and East Asian members of section *Ilex* (Simeone et al. 2018). Representative sequence: HE591247

**H13** = Derived haplotype of the ‘Cerris-Ilex’ lineage collecting GenBank sequences of Q. cerris and Q. look from Isreal and Lebanon (Simeone et al. 2018). Representative sequence: LT963528

**H14** = Derived haplotype of the ‘Cerris-Ilex’ lineage, including *Q. cerris* and *Q. libani* from Turkey (LT963521, HE591294)

**H15** = *Quercus ilex* from Italy (LT222199), belonging to the ‘Euro-Med’ lineage of *Quercus* sect. *Ilex* (sub-lineage IV; Vitelli et al. 2017)

**H16** = several accessions of *Q. ilex* and *Q. coccifera* from France, Italy, Algeria and the Balkans, belonging to the ‘Euro-Med’ lineage of *Quercus* sect. *Ilex* (sub-lineage IV; Vitelli et al. 2017). Representative sequence: HE591264

**H17** = *Quercus alnifolia* from Cyrpus (LM652764, HE591298), *Q. coccifera* from Turkey and Lebanon (LM652815, LT222159, LT222160), belonging to the WAHEA lineage of *Quercus* section *Ilex* (Vitelli et al. 2017)

**H18** = *Quercus coccifera* from Israel and Lebanon (HE591259, LT222161), belonging to the WAHEA lineage of *Quercus* section *Ilex* (Vitelli et al. 2017)
